# A Case of an Autoimmune Blistering Disease: Pemphigus Vulgaris

**DOI:** 10.7759/cureus.61679

**Published:** 2024-06-04

**Authors:** Corinne Ricci, Blake Van Noord, Aaron Burch, McKenzie Tibbs

**Affiliations:** 1 Dermatology, Rocky Vista University College of Osteopathic Medicine, Englewood, USA; 2 Dermatology, LewisGale Medical Center, Salem, USA; 3 Dermatology, LewisGale Hospital Montgomery HCA Virginia Health System, Blacksburg, USA

**Keywords:** clinical presentation of pv, treatment choices, autoimmune blistering skin disease, autoimmune skin disorders, pemphigus vulgaris

## Abstract

Pemphigus vulgaris is a rare autoimmune disorder characterized by the formation of intraepithelial blisters that clinically appear as erosions and flaccid bullae on the skin and mucus membranes. Herein, we report a case of pemphigus vulgaris in an elderly male. He was initially misdiagnosed by his primary care provider and given topical lidocaine and acetaminophen with hydrocodone, without improvement in symptoms. This delay in treatment caused a worsening of his condition. The patient presented to our dermatology office two months after his primary care visit and reported worsening blisters and pain. Clinically he presented with flaccid bullae, crusted erosions, and erythematous plaques on the chest, back, abdomen, arms, and legs, and a tender oral ulcer. Two punch biopsies were obtained and sent for direct immunofluorescence and routine histology. The biopsy results confirmed the diagnosis of pemphigus vulgaris. Our patient achieved clearance after four weeks of oral prednisone and maintained clearance after a slow prednisone taper and the addition of mycophenolate mofetil 1g twice daily. We aim to bring awareness of the clinical presentation and treatment regimen of pemphigus vulgaris to prevent misdiagnosis and delayed care.

## Introduction

Pemphigus designates a group of rare, life-threatening autoimmune blistering disorders that affect the skin and mucous membranes. The disease is characterized by autoantibodies that target intracellular adhesion molecules, resulting in acantholysis, formation of intraepithelial blisters, and the clinical appearance of erosions and flaccid bullae [1]. Pemphigus vulgaris (PV) is the most common clinical variant with an estimated incidence of one to five cases per million individuals annually. There is a higher occurrence of cases among Ashkenazi Jews and individuals from the Middle East, India, and Southeast Europe [2]. PV affects males and females equally with the average onset between the fourth and sixth decade of life [2]. In comparison to the general population matched for age and sex, PV shows a 2.6-fold rise in mortality [3]. The etiology is not thoroughly understood, but it is likely multifactorial, involving a combination of genetic predisposition and environmental factors. Prompt diagnosis with treatment can limit complications of disease; however, it is often mistaken for other diseases, particularly if only oral lesions are present [4]. Treatment typically consists of systemic corticosteroids and/or rituximab depending on disease severity [4]. In this report, we present a case of pemphigus vulgaris that was initially misdiagnosed, resulting in the worsening of the disease before receiving appropriate treatment. We aim to increase awareness of presentation to primary care providers and explore current therapies.

## Case presentation

An 82-year-old male presented as a referral for a generalized rash involving the chest, back, arms, legs, and oral mucosa. The rash began two months prior to our visit with reported associated blistering, pain and pruritus. Initially, he visited his primary care provider and received prescriptions for narcotics, topical lidocaine, and topical betamethasone without relief of symptoms. Subsequently, he was referred to dermatology for worsening blisters and pain. The patient did not exhibit any constitutional symptoms and denied previous dermatologic history, preceding infections, introduction of new personal care products, or new medications. His medication list included simvastatin, aspirin, isosorbide mononitrate, amlodipine, fluticasone propionate, bupropion, clonazepam, apixaban, and tamsulosin.

**Figure 1:**
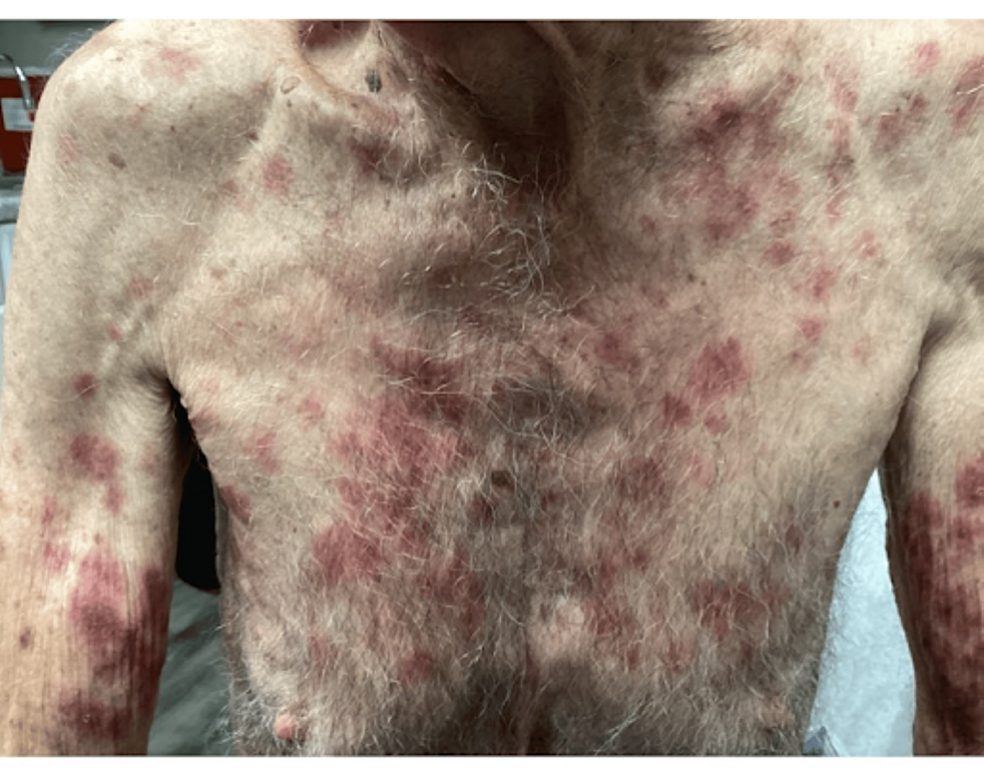
Patient Presentation - Chest and Arms Flaccid bullae, crusted erosions, and erythematous plaques were present on the chest and arms

**Figure 2:**
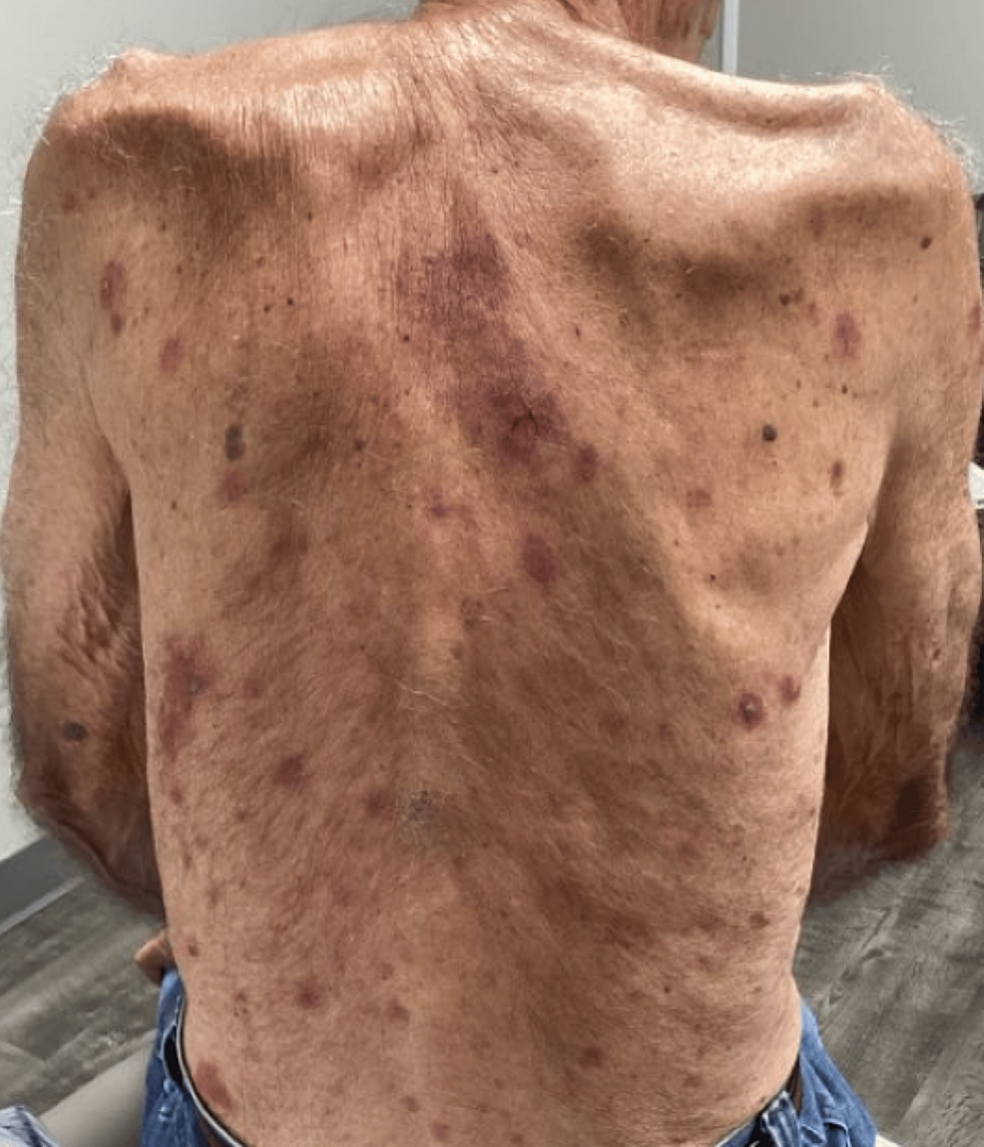
Patient Presentation - Back and Posterior Arms Flaccid bullae, crusted erosions, and erythematous plaques dispersed on the patient's back and posterior arms

Examination revealed flaccid bullae, crusted erosions, and erythematous plaques on the chest, back, abdomen, arms, and legs (Figures 1-2).

**Figure 3:**
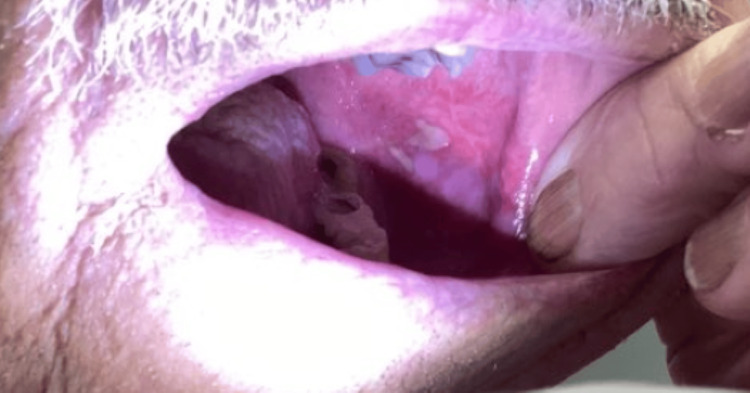
Patient Presentation - Intraoral Examination An isolated oral ulcer was observed on the left buccal mucosa.

Additionally, an isolated, tender oral ulcer was observed on the left buccal mucosa (Figure 3).

Differential diagnoses included pemphigus vulgaris, pemphigus foliaceus, bullous impetigo, lupus erythematosus, and Stevens-Johnston syndrome. Two punch biopsies were obtained on his upper back, lesional and perilesional, and sent for routine histology and direct immunofluorescence (DIF), respectively.

**Figure 4:**
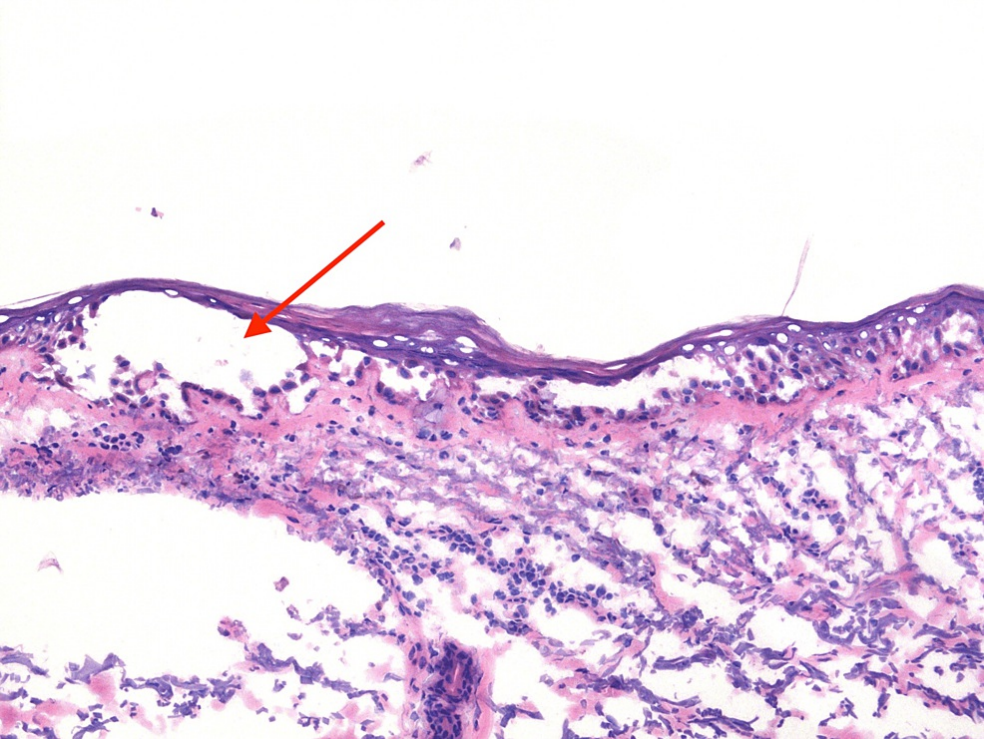
H&E Staining Performed on the Punch Biopsy Specimen Denuded epidermis with acantholysis and cleft formation (red arrow). Dermal eosinophils are present. H&E: hematoxylin and eosin

Pathology results demonstrated a denuded epidermis with dermal eosinophils with a superficial dermis featuring fibrin, degenerative debris, and hemosiderin (Figure 4).

**Figure 5:**
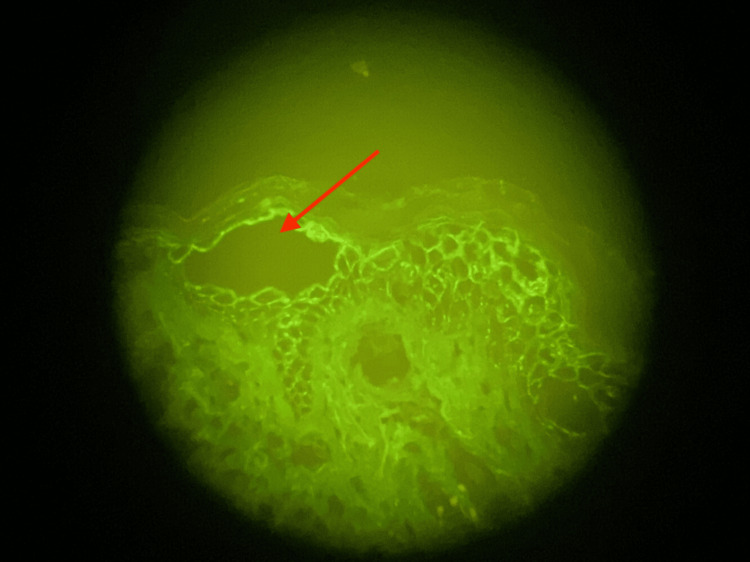
Direct Immunofluorescence of Perilesional Biopsy Perilesional skin positive for C3 and IgG staining intracellularly in the epidermis (red arrow).

Deeper dermis contained superficial and deep, perivascular and interstitial, mixed inflammatory infiltrate composed of lymphocytes and eosinophils. DIF demonstrated positive intercellular deposition of IgG and C3 across the epidermis (Figure 5).

The biopsy results confirmed the diagnosis of pemphigus vulgaris. Oral prednisone was initiated at 0.5mg/kg/day, and the patient was advised to begin vitamin D and calcium supplementation. Clearance was achieved by the patient at Week 4 of treatment, with no new cutaneous or mucosal bullae observed.

## Discussion

Herein, we report a case of pemphigus vulgaris, a rare autoimmune blistering disease characterized clinically by erosions and/or flaccid bullae affecting mucous membranes and the skin [1]. In approximately 70% of cases, patients exhibit painful erosions of the oral mucosa, while erosions of the genital mucosa are observed in 20% of cases [2]. In contrast, our patient initially presented to his primary care provider with widespread bullae and erosions on the skin with minimal oral involvement. The oral mucous membranes commonly affected included the gingiva, floor of the mouth, hard and soft palate, posterior pharynx, and labial mucosa [5]. These lesions are often painful, persist for extended periods of time, and are frequently misdiagnosed as aphthous ulcers, herpes stomatitis, or candidiasis [6]. When skin is affected, patients more commonly present with flaccid blisters, erosions, and weeping lesions distributed across the trunk, flexural regions, extremities, face, and scalp [2,5]. This distribution of skin lesions was observed in our patient during the physical examination. Active cutaneous blisters are rarely identified due to the fragility of the blister.

Pemphigus vulgaris is associated with IgG autoantibodies that specifically target key components of desmosomes, namely desmoglein 1 and 3. Desmoglein is a transmembrane glycoprotein crucial for maintaining cell-to-cell adhesion within desmosomes [2]. When the IgG autoantibody binds to desmoglein, there is a loss of cohesion between the epidermal keratinocytes, leading to intraepidermal blisters [2]. The histological examination of the biopsy specimens taken from our patient played a key role in the diagnosis by complementing the associated clinical findings. For the diagnosis of PV, guidelines recommend obtaining a biopsy from the periphery of a fresh vesicle or bulla to be sent for routine histology. For direct immunofluorescence (DIF), perilesional skin is preferred. Histologically, PV may exhibit characteristic features such as intraepithelial separation involving acantholysis formed in the suprabasal region, preserved basal keratinocytes attached to the basement membrane resembling a row of tombstones, and limited inflammatory infiltrates [1]. The pathology report from our patient's biopsy also detected a mixed inflammatory infiltrate consisting of lymphocytes and eosinophils. While the presence of eosinophils is highly sensitive for these disorders, the ultimate diagnosis relies on other findings, given the low specificity [7]. Direct immunofluorescence (DIF) performed on the perilesional biopsy will show IgG and C3 intracellular deposits, which were identified on our patient’s biopsy [1].

The primary goal of treatment is to facilitate the healing of existing lesions, followed by maintaining remission once cleared. The choice of treatment is determined by the severity of the disease and the patient’s comorbidities. Our patient achieved clearance after one month of prednisone and maintained clearance after a slow taper and the addition of mycophenolate mofetil 1g taken twice daily. Pemphigus Disease and Area Index (PDAI) and the Autoimmune Bullous Skin Intensity and Severity Score (ABSIS) are two clinical scoring tools to grade disease severity [8,9]. Historically, systemic corticosteroids have been the treatment of choice for all forms of pemphigus vulgaris. However, due to the absence of large, randomized controlled trials, recommendations largely rely on expert opinion. Corticosteroids are still widely considered first-line therapy for mild disease, with a recommended dosing of 0.5mg/kg/day [10]. In moderate-to-severe disease, the typical dosage range is 1-2 mg/kg/day. Moreover, topical corticosteroids alone may suffice for patients with mild disease and limited lesions [10].

The increased mortality rate of PV (ranging from 5% to 30% at different follow-up periods) has been associated with adverse effects resulting from prolonged use of immunosuppressants and corticosteroids, which can lead to severe infections [3,5]. Relapses manifest in approximately 50% of patients treated with corticosteroids alone; however, since the advent of rituximab as first-line therapy, remission was achieved in 70% of patients within six months and 90% remission after two years [11,12]. In 2018, rituximab became the first therapy approved for moderate-to-severe pemphigus by the US Food and Drug Administration. Rituximab is a monoclonal antibody targeting CD20-positive B lymphocytes [10]. Currently, it is recommended as first-line therapy for moderate-to-severe pemphigus according to several society guidelines, both with and without corticosteroids [4,10]. Dosing is typically four infusions of 375 mg/m2 one week apart or two infusions of 1000 mg 2 weeks apart, with the latter more commonly used and more cost-effective [13]. Adjunctive therapies most commonly include azathioprine and mycophenolate mofetil, although cyclophosphamide, IVIG, and immunoadsorption have also been used [4]. Once the disease is controlled, characterized by the healing of approximately 80% of skin and mucosal lesions without the emergence of new lesions for at least two weeks, a gradual reduction of corticosteroids should be commenced. Faster tapers are advisable if the patient is simultaneously undergoing rituximab therapy [10]. If relapse occurs, the patient should return to taking the lowest possible dose of corticosteroids that can control the disease or consider adding an immunosuppressant [4].

## Conclusions

In conclusion, in the era predating corticosteroids, pemphigus vulgaris posed a fatal threat, with a majority of patients succumbing within five years of disease onset. The introduction of systemic corticosteroids and immunosuppressive therapies has significantly enhanced the prognosis for patients. Contemporary treatment approaches not only extend disease-free intervals but also inspire optimism for future therapies, which ushers in a new era of improved patient outcomes and quality of life. This case highlights the importance for primary care providers to be well-versed in the possible manifestations of pemphigus vulgaris and utilize clinical presentation to guide treatment decisions and appropriate specialty referrals. 
